# Examining the Relationship and Prognostic Significance of Cell-Free DNA Levels and the PSMA-Positive Tumor Volume in Men with Prostate Cancer: A Retrospective–Prospective [^68^Ga]Ga-PSMA-11 PET/CT Study

**DOI:** 10.2967/jnumed.123.266158

**Published:** 2024-01

**Authors:** Kilian Kluge, Holger Einspieler, David Haberl, Clemens Spielvogel, Stefan Stoiber, Chrysoula Vraka, Laszlo Papp, Sabine Wunsch, Gerda Egger, Gero Kramer, Bernhard Grubmüller, Shahrokh Shariat, Marcus Hacker, Lukas Kenner, Alexander Haug

**Affiliations:** 1 Division of Nuclear Medicine, Department of Biomedical Imaging and Image-Guided Therapy, Medical University of Vienna, Vienna, Austria;; 2 Christian Doppler Laboratory for Applied Metabolomics, Medical University of Vienna, Vienna, Austria;; 3 Department of Pathology, Medical University of Vienna, Vienna, Austria;; 4 Center for Medical Physics and Biomedical Engineering, Medical University of Vienna, Vienna, Austria;; 5 Department of Urology, Medical University of Vienna, Vienna, Austria;; 6 Department of Urology and Andrology, University Hospital Krems, Krems, Austria;; 7 Karl Landsteiner University of Health Sciences, Krems, Austria;; 8 Karl Landsteiner Institute of Urology and Andrology, Vienna, Austria;; 9 Department of Urology, University of Texas Southwestern Medical Center, Dallas, Texas;; 10 Division of Urology, Department of Special Surgery, University of Jordan, Amman, Jordan;; 11 Department of Urology, Second Faculty of Medicine, Charles University, Prague, Czech Republic; and; 12 Department of Urology, Weill Cornell Medical College, New York, New York

**Keywords:** liquid biopsy, cell-free DNA, prostate cancer, PSMA, PET/CT

## Abstract

Functional imaging with prostate-specific membrane antigen (PSMA) ligands has emerged as the standard imaging method for prostate cancer (PCA). In parallel, the analysis of blood-derived, cell-free DNA (cfDNA) has been shown to be a promising quantitative biomarker of PCA aggressiveness and patient outcome. This study aimed to evaluate the relationship and prognostic value of cfDNA concentrations and the PSMA-positive tumor volume (PSMA-TV) in men with PCA undergoing [^68^Ga]Ga-PSMA-11 PET/CT imaging. **Methods:** We recruited 148 men with histologically proven PCA (mean age, 70.7 ± 7.7 y) who underwent [^68^Ga]Ga-PSMA-11 PET/CT (184.9 ± 18.9 MBq) and blood sampling between March 2019 and August 2021. Among these, 74 (50.0%) had hormone-sensitive PCA and 74 (50.0%) had castration-resistant PCA (CRPC). All patients provided written informed consent before blood sample collection and imaging. The cfDNA was extracted and quantified, and PSMA-expressing tumor lesions were delineated to extract the PSMA-TVs. The Spearman coefficient assessed correlations between PSMA-TV and cfDNA concentrations and cfDNA’s relation with clinical parameters. The Kruskal–Wallis test examined the mean cfDNA concentration differences based on PSMA-TV quartiles for significantly correlated patient groups. Log-rank and multivariate Cox regression analyses evaluated the prognostic significance of high and low cfDNA and PSMA-TV levels for overall survival. **Results:** Weak positive correlations were found between cfDNA concentration and PSMA-TV in the overall group (*r* = 0.16, *P* = 0.049) and the CRPC group (*r* = 0.31, *P* = 0.007) but not in hormone-sensitive PCA patients (*r* = −0.024, *P* = 0.837). In the CRPC cohort, cfDNA concentrations significantly differed between PSMA-TV quartiles 4 and 1 (*P* = 0.002) and between quartiles 4 and 2 (*P* = 0.016). Survival outcomes were associated with PSMA-TV (*P* < 0.0001, *P* = 0.004) but not cfDNA (*P* = 0.174, *P* = 0.12), as per the log-rank and Cox regression analysis. **Conclusion:** These findings suggest that cfDNA might serve as a biomarker of advanced, aggressive CRPC but does not reliably reflect total tumor burden or prognosis. In comparison, [^68^Ga]Ga-PSMA-11 PET/CT provides a highly granular and prognostic assessment of tumor burden across the spectrum of PCA disease progression.

## Visual Abstract

**Figure:**
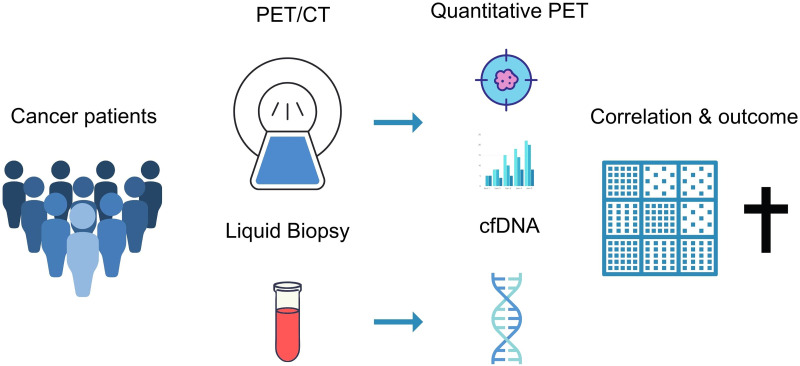

Despite substantial diagnostic and therapeutic innovations in recent years, prostate cancer (PCA) remains a leading cause of cancer-related mortality in men (1).

As PCA progresses from initially localized and hormone-sensitive PCA (hsPC) to progressively metastatic and castration-resistant PCA (CRPC)—a transition predominantly characterized by a loss of reliance on gonadal androgen signaling (2*,*^3^)—periodic reassessment of tumor progression is critical to enable appropriate therapy adjustments (4).

Hybrid imaging with PET/CT using prostate-specific membrane antigen (PSMA) ligands (5) has emerged as the diagnostic imaging gold standard using highly sensitive and specific radiotracers not reliant on the Warburg effect (6), as they enable highly accurate PCA staging (7*,*^8^), frequently leading to changes in disease management (9).

In parallel, the analysis of blood-derived, cell-free DNA (cfDNA) has recently gained scientific traction (10) in oncology because of its minimally invasive nature and the wealth of predictive and prognostic information it is able to provide. In healthy individuals, cfDNA is believed to originate primarily from apoptosis or necrosis of hematopoietic cells (11*,*^12^). In cancer patients, tumor and tumor-microenvironment–constituting cells also have been shown (13*,*^14^) to shed DNA (circulating-tumor DNA) into the bloodstream by apoptosis, necrosis (15), and even active secretion (16). As a result, supraphysiologic cfDNA concentrations are frequently observed in cancer patients (17–19).

Genomic and epigenomic interrogations of circulating-tumor DNA using approaches based on polymerase chain reaction or next-generation sequencing enable in-depth profiling of PCA biology, evolution, and prognostic trajectory (20–23). However, simple quantification of cfDNA levels was shown to be a cost-effective, prognostic, and predictive biomarker in several studies (24*,*^25^), including 2 multicenter, taxane-evaluating phase III chemotherapy trials (24). Likewise, qualitative and semiquantitative analysis of PSMA-ligand PET/CT imaging has repeatedly been demonstrated to yield valuable biomarkers of disease outcome and therapy responses (26).

To date, the relationship between functional imaging assessments of tumor burden and cfDNA levels has been investigated only using [^18^F]fluorocholine PET/CT imaging (27). However, in the setting of [^68^Ga]Ga-PSMA-HBED-CC ([^68^Ga]Ga-PSMA-11) PET/CT, the association between cfDNA levels and PSMA-positive tumor volume (PSMA-TV), as well as their comparative prognostic value, remains unexplored.

We hypothesized that cfDNA levels correlate with functionally imaged tumor volumes and that both methods yield survival outcome–associated information.

This study aimed to assess the relationship between cfDNA levels and PSMA-TV, as well as their prognostic value, in men with PCA undergoing [^68^Ga]Ga-PSMA-11 PET/CT imaging.

## MATERIALS AND METHODS

### Study Design

**FIGURE 1.**
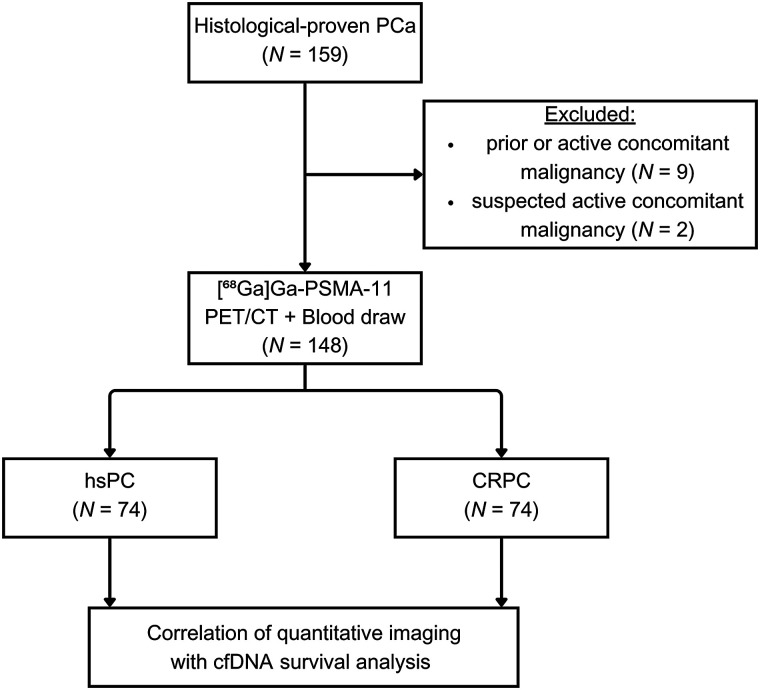
CONSORT (Consolidated Standards of Reporting Trials) diagram.

In this single-center study, PCA patients referred for [^68^Ga]Ga-PSMA-11 PET/CT imaging at the Medical University of Vienna between March 2019 and August 2021 were prospectively recruited. Only patients with histologically proven PCA were included, excluding those with other active or previous malignancies (Fig. 1). Blood samples were collected for cfDNA analysis after obtaining written informed consent. This study was approved by the ethics committee of the Medical University of Vienna (approval 1649/2016).

Clinical data were gleaned retrospectively from the medical records. Follow-up and overall survival (OS) data were sourced from Statistics Austria (censoring date, May 12, 2023). The primary endpoints of this study were, first, the relationship between cfDNA concentrations and PSMA-TV and, second, the prognostic value of cfDNA and PSMA-TV levels stratified according to high- and low-level groups. The secondary endpoint was the association of cfDNA concentration with PSMA-TV quartiles, in case the first primary endpoint was met (supplemental methods; supplemental materials are available at http://jnm.snmjournals.org).

### Plasma Sample Collection and Storage, DNA Extraction, and Quantification

Blood samples were collected in cfDNA BCT tubes (Streck Inc.) before tracer injection and centrifuged to remove any cellular debris. The resulting plasma was stored at −80°C (supplemental methods). The QIAamp Circulating Nucleic Acid Kit (Qiagen) was used to extract cfDNA from plasma according to the manufacturer’s instructions, and the resultant cfDNA was stored at −20°C for further analysis. cfDNA was quantified on a Fragment Analyzer (Agilent) using an HS Next-Generation Sequencing Fragment Kit (Agilent). PROSize software (version 2.0; Agilent) analyzed the electropherograms and quantified cfDNA concentration, expressed as ng/μL of elution volume (example electrophoresis reports are shown in the supplemental materials).

### Imaging Protocol and Image Analysis

All scans were performed using a Biograph TruePoint PET/CT scanner (Siemens Healthineers), with patients receiving an intravenous injection of a mean of 184.9 MBq (±18.9 SD) of [^68^Ga]Ga-PSMA-11. One-hour after injection, static whole-body scans were obtained from the skull base to the upper femur.

First, CT scans were acquired, followed by PET scans, which were reconstructed using a point-spread-function–based algorithm.

Two nuclear medicine physicians analyzed the images using Hybrid 3-dimensional software (version 4.0.0; Hermes Medical Solutions), manually delineating all PSMA-expressing primary and secondary tumor lesions. The PSMA-TV was extracted from all delineated lesions analogously to the calculation of the metabolic tumor volume, and the dominant tumor fraction, contributing most to overall PSMA-TV, was defined (supplemental methods).

### Statistical Analysis

Continuous variables are expressed as mean (±SD), and discrete outcomes are expressed as absolute and relative (%) frequencies.

The Shapiro–Wilk test assessed the normality of variables; correlations of variables were assessed using the Spearman coefficient; heteroskedasticity was checked with the Levene test. The difference in mean cfDNA levels according to PSMA-TV quartiles was assessed using the Kruskal–Wallis test, facultatively followed by the Dunn-Bonferroni post hoc test. χ^2^ testing assessed associations between dominant tumor lesion fraction and PSMA-TV quartiles. The Kaplan–Meier test estimated OS probabilities; the log-rank test compared survival distributions between groups with high and low cfDNA and PSMA-TV (cutoff, respective median levels). Multivariate Cox regression analysis evaluated the relationship between OS and the binary variables cfDNA concentration and PSMA-TV, which were checked for multicollinearity and proportional hazards with the Belsley–Kuh–Welsch technique and Schoenfeld residuals, respectively. The α-risk was set at 5% for all analyses, conducted with EasyMedStat software (version 3.24) (supplemental methods).

## RESULTS

### Clinical Cohort

In total, 148 patients (age, 70.7 ± 7.7 y; prostate-specific antigen [PSA], 107.67 ± 454.10) with histologically proven PCA were recruited. The median follow-up duration was 19 mo (range, 0–49 mo). OS was 90.5% (95% CI, 84.4%–94.2%) at 12 mo and 87.1% (CI, 80.5%–91.6%) at 24 mo. The clinical and demographic characteristics are presented in Table 1.

**TABLE 1.** Demographic and Clinical Patient Data

Variable	hsPC (*n* = 74)	CRPC (*n* = 74)
Age at inclusion (y)	69.9 ± 7.8 (50.0–85.0)	71.5 ± 7.5 (49.0–85.0)
Tracer dose (MBq)	185.6 ± 20.7 (134.0–300.0)	184.3 ± 17.1 (149.0–263.0)
cfDNA (ng/μL)	0.745 ± 0.654 (0.0009–4.25)	1.04 ± 1.42 (0.0818–9.49)
PSMA-TV (cm^3^)	14.2 ± 76.7 (0.0–659.1)	175.5 ± 369.2 (0.0–1,597.7)
PSMA-positive lesion		
Any lesion	51 (68.9%)	64 (86.5%)
Prostate lesion	25 (33.8%)	19 (25.7%)
Lymph node lesion	26 (35.1%)	37 (50.0%)
Bone lesion	14 (18.9%)	48 (64.9%)
Organ lesion	4 (5.4%)	13 (17.6%)
Dominant fraction		
Prostate	19 (25.7%)	5 (6.8%)
Lymph node	22 (29.7%)	19 (25.7%)
Bone	8 (10.8%)	39 (52.7%)
Organ	2 (2.7%)	1 (1.4%)
PSA (ng/dL)*	24.98 ± 105.34 (0.09–761.0)	186.42 ± 618.29 (0.01–3,689.0)
Hemoglobin (g/dL)^†^	14.06 ± 1.64 (12.1–17.8)	11.96 ± 1.84 (7.8–15.4)
Lactate dehydrogenase (U/L)^‡^	201.21 ± 47.37 (149.0–312.0)	250.05 ± 229.26 (130.0–1,573.0)
Systemic therapies while PET		
Antihormonal therapies	4 (5.41%)	55 (78.57%)
Cytotoxic therapies	1 (1.35%)	3 (16.67%)
Systemic therapies after PET		
Local	24 (55.8%)	10 (23.3%)
Local + ADT	6 (14.0%)	—
ADT	9 (20.9%)	14 (32.6%)
CHT	1 (2.3%)	2 (4.7%)
CHT + ADT	1 (2.3%)	—
^ 177^Lu-PSMA	1 (2.3%)	16 (37.2%)
Study	1 (2.3%)	1 (2.3%)
Mean follow-up (mo)	19.8 ± 13.5 (0.0–47.9)	16.0 ± 14.0 (0.0–49.0)

* *n* = 14 and 11 data missing in hsPC and CRPC groups, respectively.

† *n* = 59 and 35 data missing in hsPC and CRPC groups, respectively.

‡ *n* = 60 and 36 data missing in hsPC and CRPC groups, respectively.

ADT = androgen deprivation therapy; CHT = concurrent hormone therapy.

Qualitative data are number and percentage; continuous data are mean ± SD and range. Local disease comprised prostate and seminal vesicle lesions.

### Associations of cfDNA Levels with Imaging Findings, Demographic Data, and Clinical Data

**FIGURE 2.**
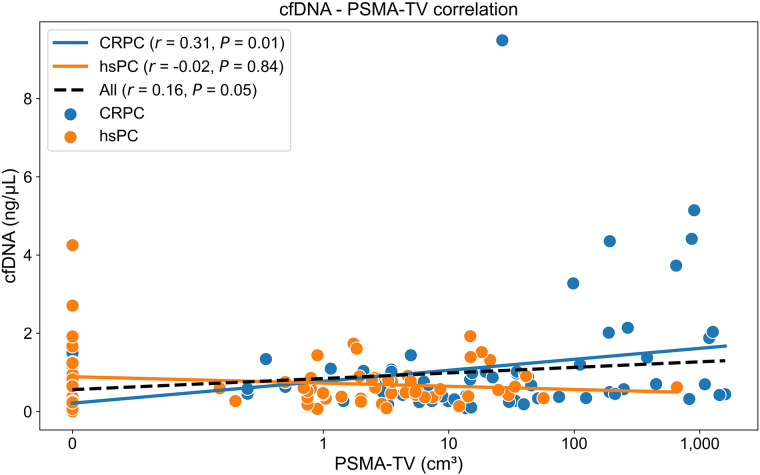
Scatterplot illustrating correlations between cfDNA concentrations and PSMA-TV in all, hsPC, and CRPC patients. PSMA-TV levels have been logarithmically transformed (log^2^ *n*) for scale comparability.

A weak positive correlation between cfDNA concentration and PSMA-TV was observed in the overall cohort (*r* = 0.16, *P* = 0.049) and the CRPC group (*r* = 0.31, *P* = 0.007) but not in the hsPC group (*r* = −0.024, *P* = 0.837) (Fig. 2).

**FIGURE 3.**
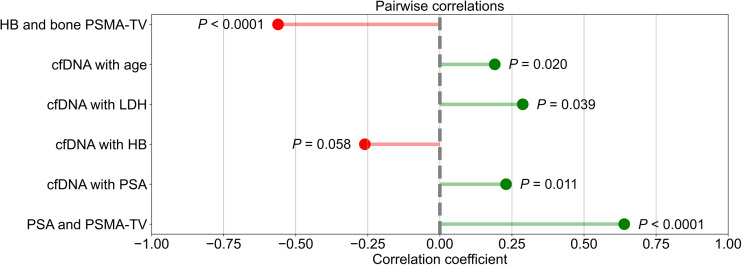
Lollipop plots illustrating correlations between cfDNA and PSMA-TV with several variables. HB = hemoglobin.

In the overall cohort, a moderate positive correlation was found between PSA level and PSMA-TV (*r* = 0.64, *P* < 0.001), and weak positive correlations were identified between cfDNA concentration and PSA (*r* = 0.23, *P* = 0.01), lactate dehydrogenase (*r* = 0.29, *P* = 0.039), and age (*r* = 0.19, *P* = 0.02). cfDNA and hemoglobin concentrations (*r* = −0.26, *P* = 0.058) showed a weak negative trend, whereas there was a moderate, significant negative correlation between bone PSMA-TV and hemoglobin (*r* = −0.56, *P* < 0.001) (Fig. 3).

### Comparison of cfDNA Levels According to PSMA-TV Quartiles

Median cfDNA concentrations across PSMA-TV quartiles did not differ significantly (*P* = 0.095) in the overall cohort, whereas there were significant differences in the CRPC group (*P* = 0.012).

**FIGURE 4.**
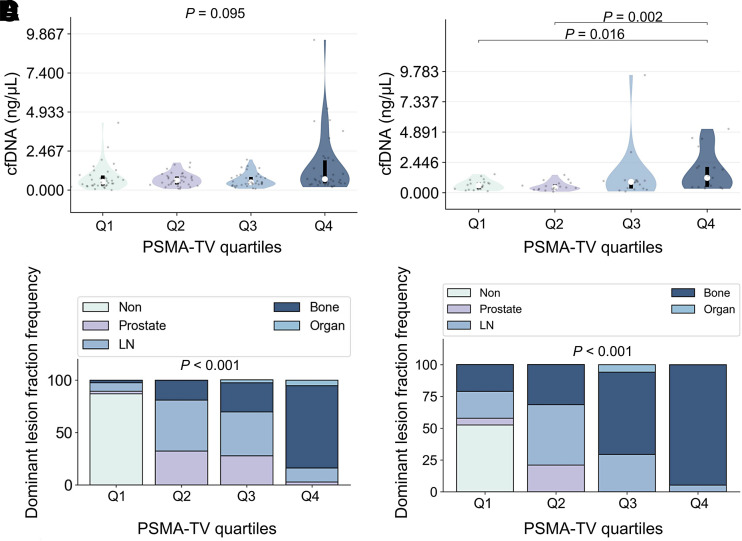
Violin plots showing relationship between cfDNA concentrations and PSMA-TV quartiles for all (A) and CRPC (B) patients. Bar plots illustrate frequency of dominant tumor lesion fraction according to PSMA-TV quartiles in all (C) and CRPC (D) patients. LN = lymph node.

Pairwise post hoc analyses revealed significant cfDNA concentration differences for PSMA-TV quartile 4 (Q4) versus quartile 1 (Q1) (*P* = 0.016) and Q4 versus quartile 2 (Q2) (*P* = 0.002) (Fig. 4; Tables 2 and 3).

**TABLE 2.** Unpaired Kruskal–Wallis Distribution of PSMA-TV Quartiles and Corresponding cfDNA Concentrations for Overall and CRPC Patient Groups

			cfDNA								
Group	Quartile	PSMA-TV range (cm^3^)	*n*	Mean	Mean 95% CI	SD	Minimum	Q1	Median	Q3	Maximum
All	Q1	0–0.2	38	0.769	0.503–1.03	0.809	0.0009	0.282	0.528	0.919	4.25
	Q2	0.2–3.5	37	0.679	0.54–0.818	0.417	0.0674	0.355	0.61	0.86	1.73
	Q3	3.5–23.6	36	0.632	0.483–0.781	0.44	0.0818	0.352	0.488	0.83	1.92
	Q4	23.6–1,597.7	37	1.48	0.856–2.11	1.88	0.183	0.421	0.694	1.88	9.49
CRPC	Q1	−1.0–3.0	19	0.625	0.431–0.818	0.402	0.126	0.284	0.581	0.822	1.49
	Q2	3.0–14.0	19	0.516	0.345–0.687	0.354	0.0818	0.265	0.403	0.665	1.44
	Q3	14.0–108.0	17	1.32	0.178–2.46	2.22	0.102	0.337	0.871	0.981	9.49
	Q4	108.0–1,598.0	19	1.72	0.965–2.48	1.57	0.32	0.472	1.2	2.09	5.14

**TABLE 3.** Post Hoc Adjusted Pairwise Table Displaying cfDNA Differences Between PSMA-TV Quartiles in CRPC Group

Quartile	Median difference	Mean difference	Mean difference in 95% CI	*P*
Q1 vs. Q2	0.18	0.11	−0.23 to 0.44	0.451
Q1 vs. Q3	−0.29	−0.7	−2.11 to 0.71	0.38
Q1 vs. Q4	−0.62	−1.1	−2.11 to −0.086	0.016
Q3 vs. Q2	0.47	0.8	−0.6 to 2.21	0.107
Q4 vs. Q2	0.8	1.21	0.2 to 2.21	0.002
Q4 vs. Q3	0.33	0.4	−1.33 to 2.13	0.146

### Associations of Dominant Tumor Lesion Fraction with PSMA-TV Quartiles

In the overall cohort, the PSMA-TV distribution based on dominant tumor lesion fractions differed significantly (*P* < 0.001). For prostate, Q1 was 2.6%, Q2 was 32.4%, quartile 3 (Q3) was 27.8%, and Q4 was 2.7%. For lymph node, Q1 was 7.9%, Q2 was 48.7%, Q3 was 41.7%, and Q4 was 13.5%. For bone, Q1 was 2.6%, Q2 was 18.9%, Q3 was 27.8%, and Q4 was 78.4%. For organ, Q1 was 0.0%, Q2 was 0.0%, Q3 was 2.8%, and Q4 was 5.4%.

In the CRPC group, the PSMA-TV distribution according to the dominant tumor lesion fractions differed significantly (*P* < 0.001). For prostate, Q1 was 5.3%, Q2 was 21.1%, and Q3–Q4 was 0.0%. For lymph node, Q1 was 21.1%, Q2 was 47.4%, Q3 was 29.4%, and Q4 was 5.3%. For bone, Q1 was 21.1%, Q2 was 31.6%, Q3 was 64.7%, and Q4 was 94.7%. For organ, Q1 was 0.0%, Q2 was 0.0%, Q3 was 5.9%, and Q4 was 0.0% (Fig. 4).

### OS Analysis

**FIGURE 5.**
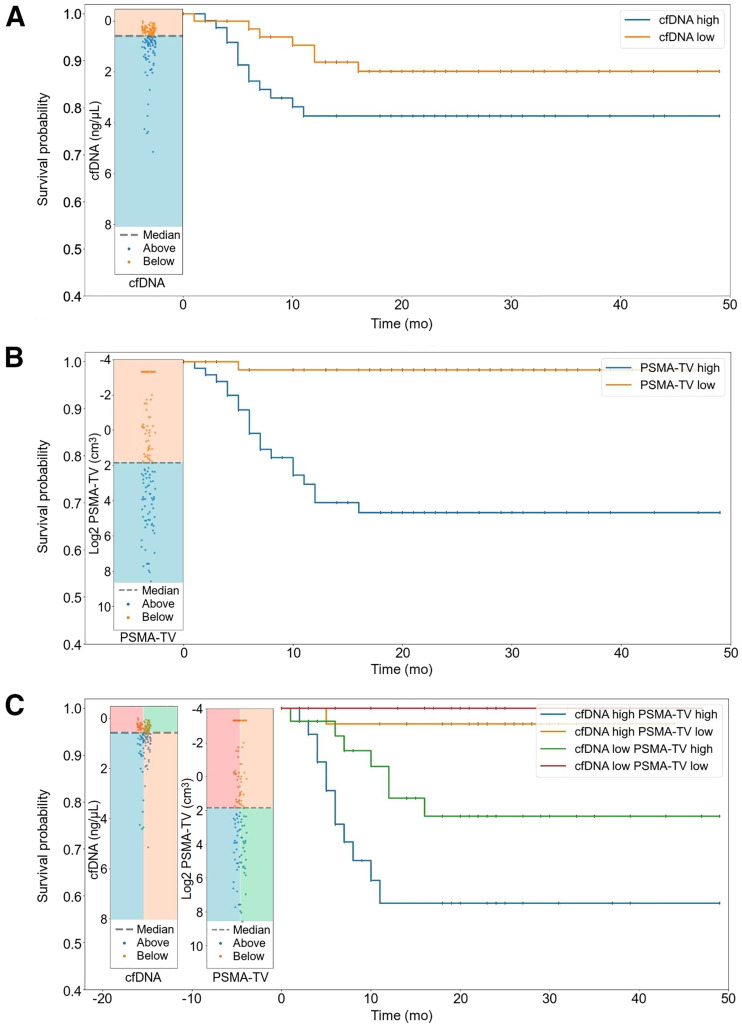
Kaplan–Meier curves illustrating survival probabilities between high- and low-cfDNA groups (A), high- and low–PSMA-TV groups (B), and compound-stratified groups (C).

Survival distributions of the cfDNA high (12 mo, 86.1%; CI, 75.7–92.3) and low (12 mo, 94.7%; CI, 86.4–98.0) groups did not significantly differ (*P* = 0.174). However, there was a significant difference in survival distributions between PSMA-TV high (12 mo, 82.1%; CI, 71.6–89.0) and low (12 mo, 100.0%; CI, 100.0–100.0) groups (*P* < 0.0001). Likewise, there was a significant difference in survival distributions between the cfDNA high–PSMA-TV high (12 mo, 73.0%; CI, 55.6–84.4), cfDNA high–PSMA-TV low (12 mo, 100.0%; CI, 100.0–100.0), cfDNA low–PSMA-TV high (12 mo, 90.2%; CI, 76.1–96.2), and cfDNA low–PSMA-TV low (12 mo, 100.0%; CI, 100.0–100.0) groups (*P* = 0.0003). In the multivariate Cox regression analysis, there were significant hazard differences between the PSMA-TV high (hazard ratio, 18.89; CI, 2.52–141.68) and low (hazard ratio, 0.0529; CI, 0.00706–0.397) groups (*P* = 0.004) but not between the cfDNA high (hazard ratio, 2.12; CI, 0.833–5.38) and low (hazard ratio, 0.472; CI, 0.186–1.2) groups (*P* = 0.12) (Table 4; Fig. 5).

**TABLE 4.** Multivariate Cox Regression of Relationship Between OS and Binary Explanatory Variables cfDNA and PSMA-TV

Variable	Hazard ratio	95% CI	*P*
cfDNA group			0.12
Low	0.472	0.186–1.2	
High	2.12	0.833–5.38	
PSMA-TV group			0.004
High	18.89	2.52–141.68	
Low	0.0529	0.00706–0.397	

## DISCUSSION

Over the last decade, the arsenal of minimally invasive methods to assess PCA and its trajectory has vastly expanded. Functional imaging using PSMA-ligand PET/CT has been shown to stage PCA with unprecedented, disease management–changing detection rates (7*,*^8^*,*^28^*,*^29^) and to yield prognostic and predictive information on therapy responses to local and systemic approaches alike (26). In parallel, the quantification of blood-derived cfDNA has been shown repeatedly to be a valuable biomarker of PCA aggressiveness and response to taxane-based chemotherapies (24*,*^25^).

In this study, we investigated the relationship between cfDNA concentration and PSMA-TV as assessed by [^68^Ga]Ga-PSMA-11 PET/CT imaging in patients with PCA according to their castration status to evaluate to what extent these methods yield associated measures of tumor burden. Furthermore, we aimed to compare the prognostic value of cfDNA and PSMA-TV measures in terms of OS.

Our findings revealed a weak positive correlation between cfDNA concentrations and PSMA-TV in the overall cohort and the CRPC group. In contrast, neither a significant nor a trending correlation was observed in hsPC patients.

Interestingly, the overall correlation between cfDNA levels and PSMA-TV appeared to be driven primarily by high-volume disease in the CRPC subgroup (Fig. 2), which was composed mainly of osseous metastases (Fig. 4D). This hypothesis is corroborated by the observed significant differences in the CRPC group between high-volume Q4 disease and low-volume Q1 (*P* = 0.016) and Q2 (*P* = 0.002) disease in conjunction with the nonsignificant cfDNA differences in the overall cohort between any PSMA-TV quartiles. Several authors (17*,*^30^) have described the same dependency of cfDNA levels and high-volume, advanced disease. Chen et al. (17) investigated whether cfDNA concentrations and DNA fragment lengths could differentiate between localized and advanced PCA and reported that cfDNA concentrations were elevated in metastatic CRPC patients in comparison to localized disease but did not significantly differ between localized disease and healthy controls. In line with these findings, we did not observe a significant or tangibly trending association in the hsPC subgroup.

We assume that this observation is caused by the combination of 2 factors. First, the hsPC subgroup had a substantially lower tumor burden than the CRPC subgroup (mean PSMA-TV: hsPC, 14.16 ± 76.68; CRPC, 175.54 ± 369.15), missing the high-volume proportion that appeared to drive the association in the CRPC subgroup. Second, the transition from hsPC to CRPC, which is mediated through various changes in the genetic and posttranscriptional profiles of PCAs and alternative modes of androgen biosynthesis (2*,*^3^), is clinically associated with more aggressive behavior and higher rates of proliferation. As cfDNA is believed to be shed into the bloodstream by hematopoietic (11*,*^12^) and tumor cells (14*,*^15^) through apoptosis and necrosis, this might explain why cfDNA concentrations and PSMA-TV correlate weakly only in our CRPC cohort, as they might more readily outgrow their blood supply.

In conclusion, we reason that cfDNA levels did not correlate with PSMA-TV in the hsPC group because of their less proliferative nature and lower disease volumes, whereas the weak correlation in the CRPC was driven mainly by the increasingly proliferative and aggressive high-volume, bone-metastasis disease fraction.

To contextualize cfDNA levels with known prognostic demographic and clinical markers of PCA, we explored the association between cfDNA concentrations and age, PSA level, hemoglobin level, and lactate dehydrogenase level. Age and cfDNA level were weakly yet significantly associated, which has been reported by several authors (31), suggesting various mechanisms of cfDNA accumulation such as cellular senescence and decreased bloodstream clearance (32*,*^33^). PSA and cfDNA levels were also weakly associated, as reported by others (34*,*^35^), probably because of PSA’s positive relationship with PSMA-TV (Fig. 3).

Furthermore, lactate dehydrogenase levels, a common signifier of cellular destruction and a long-established negative prognostic biomarker in advanced PCA (36), correlated positively with cfDNA levels. Although we did not observe a significant correlation between hemoglobin and cfDNA levels, a negative trend was tangible. We hypothesize that this potential relationship might be caused by increased depression of hematopoietic tissue in advanced, metastatic PCA, because the dominant tumor lesion fraction was contributed mainly by osseous lesions in the higher-tumor quartiles, and there was a moderate negative correlation between bone PSMA-TV and hemoglobin levels (Fig. 3). Next, we conducted a survival analysis to assess the prognostic value of cfDNA and PSMA-TV levels by stratifying the overall cohort into high- and low-level groups using the respective median value as the cutoff. We observed significantly different survival distributions when binarily stratified into high- and low-volume PSMA-TV groups (Fig. 5B), which is in line with Has Simsek et al.’s report of a significant association of total PSMA-derived tumor volume with OS (37). However, no significant survival differences were seen in the high- and low-level cfDNA groups (Fig. 5A), which runs contrary to several studies (17*,*^24^) that reported significant associations between cfDNA levels and OS in men with CRPC undergoing taxane-based chemotherapy. To determine whether there might be a synergistic prognostic biomarker potential, we defined a compound-stratified approach combining the high- and low-cfDNA groups, which yielded no additional prognostic value (Fig. 5C) over a binary PSMA-TV stratifier. A multivariate Cox regression analysis corroborated these findings.

However, as there was a slight trend (Fig. 5B) toward lower survival probabilities in the high-cfDNA group, we hypothesize that our study might be underpowered to detect survival differences based on cfDNA concentrations, as only 19 patients had passed until the date of censoring (May 2023).

Our study had several limitations, and the results should therefore be interpreted with caution. First, since it was a single-center study, we were relying on retrospective and partly incomplete clinical data, which can negatively influence generalizability. Second, the survival analysis might have been underpowered, as only a fraction of patients had passed until censoring, which also made a castration-status-resolved survival analysis unfeasible. Third, the used metabolic tumor volume analogue PSMA-TV might not prove to be the most robust PSMA-PET parameter for survival analysis in the future, as artificial intelligence–derived measures could potentially yield more applicable metrics going forward.

However, although our study had several limitations, it is important to acknowledge its strengths. Blood sampling immediately before tracer injection ensured optimal biologic synchronicity and thereby the comparability of cfDNA and PET/CT findings. Furthermore, the nonintentional but proportional inclusion of hsPC and CRPC patients enabled a balanced, comparative analysis according to castration status. Last, we mitigated potential biases of record keeping by relying strictly on the central death registry of the national statistics service for the outcome analysis.

Future research will focus on investigating specifically the cfDNA’s tumor fraction in relationship to [^68^Ga]Ga-PSMA-11 PET/CT imaging to explore the degree of mutual information on tumor burden and prognosis, thereby potentially informing future liquid biopsy studies regarding quantitative lower limits of detection and exploring potential synergies of combined diagnostic and prognostic use.

## CONCLUSION

Our findings suggest that cfDNA might be a biomarker of advanced, aggressive CRPC but does not reliably reflect total tumor burden. In comparison, [^68^Ga]Ga-PSMA-11 PET/CT provides a highly granular and prognostic assessment of tumor burden across the spectrum of PCA disease progression.

## DISCLOSURE

Financial support was received from the Austrian Federal Ministry for Digital and Economic Affairs; the National Foundation for Research, Technology and Development; and the Christian Doppler Research Association. Siemens Healthineers provided financial and scientific support. No other potential conflict of interest relevant to this article was reported.

## Notes

KEY POINTS **QUESTION:** Are cfDNA concentrations related to [^68^Ga]Ga-PSMA-11 PET/CT PSMA-TV, and do they provide comparable prognostic information in men with PCA? **PERTINENT FINDINGS:** In this single-center [^68^Ga]Ga-PSMA-11 PET/CT imaging study comparing cfDNA levels with PSMA-TV in 148 men with histologically proven PCA (hsPC, *n* = 74; CRPC, *n* = 74), cfDNA levels were only weakly associated with high-volume CRPC and, contrary to the PSMA-TV, not associated with OS. **IMPLICATIONS FOR PATIENT CARE:** These findings suggest that cfDNA might be a biomarker of advanced, aggressive CRPC but does not reliably reflect total tumor burden or prognosis. In comparison, [^68^Ga]Ga-PSMA-11 PET/CT provides a highly granular and prognostic assessment of tumor burden across the whole spectrum of PCA disease progression.
